# The Role of Pelvic Exenteration in Cervical Cancer: A Review of the Literature

**DOI:** 10.3390/cancers16040817

**Published:** 2024-02-18

**Authors:** Ana Carla Franco Ubinha, Priscila Grecca Pedrão, Aline Cássia Tadini, Ronaldo Luis Schmidt, Marcelo Henrique dos Santos, Carlos Eduardo Mattos da Cunha Andrade, Adhemar Longatto Filho, Ricardo dos Reis

**Affiliations:** 1 Department of Gynecologic Oncology, Barretos Cancer Hospital, São Paulo 14784-400, Brazil; ronaldolsch@gmail.com (R.L.S.); oncomhs@gmail.com (M.H.d.S.); mdcarlosandrade@gmail.com (C.E.M.d.C.A.); drricardoreis@gmail.com (R.d.R.); 2 Molecular Oncology Research Center, Barretos Cancer Hospital, São Paulo 14784-400, Brazil; prigpedrao@gmail.com (P.G.P.); longatto@med.uminho.pt (A.L.F.); 3 Barretos School of Health Sciences, Dr. Paulo Prata-FACISB, Barretos 14785-002, Brazil; cassiatadini@gmail.com; 4 Medical Laboratory of Medical Investigation (LIM), Department of Pathology, Medical School, University of São Paulo, São Paulo 01246-903, Brazil; 5 Life and Health Sciences Research Institute (ICVS), School of Medicine, University of Minho, 4710-057 Braga, Portugal; 6 ICVS/3B’s—PT Government Associate Laboratory, 4710-057 Braga, Portugal; 7 ICVS/3B’s—PT Government Associate Laboratory, 4805-017 Guimarães, Portugal

**Keywords:** cervical cancer, pelvic exenteration, recurrent cervical cancer, extended pelvic resection

## Abstract

**Simple Summary:**

Cervical cancer ranks as the fourth most common malignant neoplasm among women worldwide. In low-income countries, the diagnosis usually occurs at advanced stages, with the recommendation of chemoradiotherapy. When this initial approach fails, with persistent disease or even in the context of tumor recurrence, pelvic exenteration (PE) becomes a therapeutic option to be considered. The criteria for recommending PE have evolved over time, influenced by advancements in surgical techniques and perioperative care. These developments have enhanced the capacity for extended resections and expanded treatment possibilities for patients who were previously limited to palliative measures. Through this review, we aim to provide insights and tools to facilitate the identification of patients who may benefit from such a complex procedure.

**Abstract:**

Pelvic exenteration represents a radical procedure aimed at achieving complete tumor resection with negative margins. Although it is the only therapeutic option for some cases of advanced tumors, it is associated with several perioperative complications. We believe that careful patient selection is related to better oncologic outcomes and lower complication rates. The objectives of this review are to identify the most current indications for this intervention, suggest criteria for case selection, evaluate recommendations for perioperative care, and review oncologic outcomes and potential associated complications. To this end, an analysis of English language articles in PubMed was performed, searching for topics such as the indication for pelvic exenteration for recurrent gynecologic neoplasms selection of oncologic cases, the impact of tumor size and extent on oncologic outcomes, preoperative and postoperative surgical management, surgical complications, and outcomes of overall survival and recurrence-free survival.

## 1. Introduction

The technique of pelvic exenteration (PE) was first described by Brunschwig in 1948 in a series of advanced gynecologic tumors. At that time, the procedure was performed with palliative intent, associated with various operative and postoperative complications, and a mortality rate of 23% [1].

PE is a radical surgical approach that aims to remove the tumor with free oncologic margins (R0). This usually requires en bloc resection of the pelvic organs, including the genital, urinary, and bowel tracts. It can be classified as anterior, posterior, or total, depending on which structures are resected. Even more extensive approaches may involve the removal of lateral components of the pelvis, such as muscles, nerves, bones, and great vessels, characterizing an extended pelvic resection or extended lateral endopelvic resection [2,3].

The objectives of this review are to present the most current indications for this procedure, propose criteria for case selection, offer recommendations for perioperative care, and review oncologic outcomes alongside potential associated complications. This article can also be used as a guide for case selection.

Whenever possible, this article will focus on evidence specific to cervical cancer. However, due to the limited number of studies available, information regarding gynecologic neoplasms in general will also be presented and acknowledged.

## 2. Indications and Case Selection

With the evolution of surgical techniques, PE has become a procedure ideally performed with curative intent. The criteria for its indication have also progressed historically, although they remain controversial in the literature.

In cervical cancer, the focus of this review, PE is usually considered in situations of recurrence or persistent pelvic disease after standard treatment with radiochemotherapy [4,5,6]. In these patients, other therapeutic options are rare, as the tumor usually responds poorly to chemotherapy and additional radiation is not an option due to toxicity [7]. In recent years, immunotherapy has also been used in these types of cancer, but the results are still controversial and are associated with a high level of toxicity [8].

Among gynecologic malignancies, cervical cancer is associated with the highest rates of PE [4,5,9,10,11,12]. Compared to other tumors that can be excised, such as vulvar and vaginal cancer, it also has better oncologic outcomes after the procedure [13].

Marnitz et al. pointed out the difficulty of obtaining individualized data on the outcomes of PE as primary treatment for cervical cancer, as most of the available retrospective studies use heterogeneous cohorts. The same authors also performed a retrospective analysis of 55 patients who underwent PE and 20 of whom received primary indication. In this group, considering only patients with stage IVA disease, the 5-year overall survival was 52.5% [14].

Schmidt and colleagues, in a German case series, indicated primary PE for tumors larger than 5 cm in diameter, located in the pelvis, or in the presence of a fistula from the bladder and/or rectum to the vagina. In this study, complete resection was obtained in 66% of cases, with an overall survival of 49% at 5 years and 39% at 10 years [11].

The International Federation of Gynecology and Obstetrics, in its most recent guidelines, suggests that cases of stage IVA tumors located in the central region of the pelvis may be considered for PE [15]. However, the prognosis of this approach is usually worse when compared to procedures indicated secondarily after disease recurrence [12]. Thus, chemoradiotherapy remains the standard initial treatment for locally advanced tumors [16,17].

The presence of distant metastases or peritoneal dissemination of the disease absolutely contraindicates PE with curative intent. Lymph node involvement, small bowel involvement, and lateral extension are conditions that must be analyzed individually, as we will discuss later [12,13]. It is important to understand that contraindications may vary depending on the expertise and experience of the surgical and multidisciplinary team.

In palliative care, there is poor evidence to support the practice of PE. A meta-analysis conducted by Kroon et al., published in 2019, points out that the morbidity and mortality of PE are very significant and are associated with a reduced range of overall survival [18]. Although the procedure can provide relief of some symptoms, it is very difficult to establish the real impact on improving a patient’s quality of life. Possible indications for PE in this context include extensive tumors causing pelvic pain, the presence of extensive fistulas in the vaginal, bladder, or bowel region, refractory genital or bladder bleeding, and also symptoms associated with tumor necrosis.

Returning to curative intentions, it is essential to properly select potential surgical cases. Therefore, we suggest analyzing three main parameters:

(1) Patient’s clinical condition;
(2) Tumor characteristics;
(3) Resources available at the treatment center.

**Figure 1:**
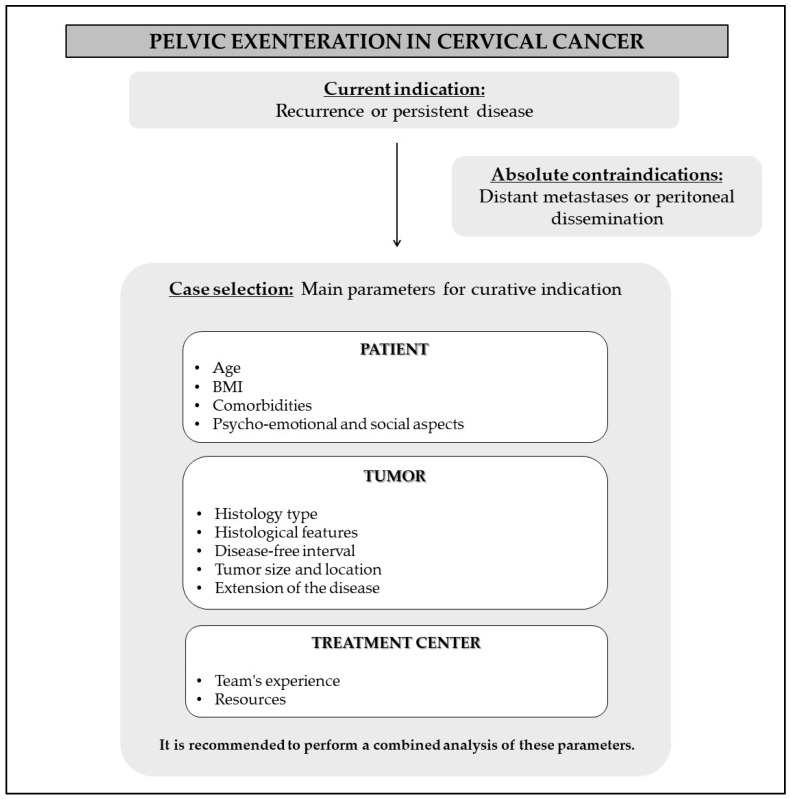
Recommendations for case selection.

Figure 1 shows a flowchart created to guide the selection of cases.

### 2.1. Assessment of the Patient’s Clinical Condition

Selecting the correct patient is extremely important as it is directly associated with two goals: (1) to reduce the number of perioperative complications, and (2) to achieve better oncologic outcomes. Criteria such as performance status, age, medical history, comorbidities, and body mass index (BMI) can be considered in combination to define if the patient is physically able to undergo this complex surgical procedure.

Age is not a parameter to be considered in an isolated way. Common sense suggests that older age is associated with worse cancer outcomes. However, several studies have found that chronological age is not a limiting factor for PE [7,14,19,20,21]. Maggioni et al., in an analysis of 106 patients undergoing PE for gynecologic cancer, showed that there was no statistical difference in survival after exenteration when comparing different age groups [5].

Schmidt et al. showed in a series of 282 cervical cancer cases undergoing EP that younger patients, aged 23–44 years, had worse 5-year survival rates (28%) compared to older groups (45 to 54 years = 46% and 55 to 79 years = 49%) [11]. On the other hand, in a retrospective analysis at Memorial Sloan Kettering Cancer Center of 71 women who were treated with PE for persistent or recurrent gynecologic cancer, Straubhar et al. reported that patients older than 62 years had worse oncologic outcomes in univariate analysis, with a negative impact on overall survival and progression-free survival [22]. Thus, with so many discrepancies, some authors suggest that physiological age should be used rather than chronological age [5,19,21].

Regarding the evaluation of medical history, it is worth mentioning the application of the Charlson Morbidity Index, described in 1987. This is a scoring scale based on a patient’s clinical conditions and possible comorbidities, with the aim of predicting the risk of complications and surgical prognosis in longitudinal studies [23]. In a retrospective analysis of 2647 cases of PE for gynecologic malignancies in the United States between 2001 and 2015, Matsuo et al. found that a high Charlson score (≥3) was associated with a higher number of postoperative adverse events, as well as longer hospital stays and higher mortality [13].

BMI is another factor that should be carefully analyzed, as obesity is directly associated with a higher incidence of postoperative complications [24]. Matsuo et al., in the same study described above, also showed that the prevalence of obesity in the gynecologic cancer population has increased significantly over the years, accompanied by a subsequent increase in the rates of surgical complications, length of hospital stay, and cost of treatment [13]. Beyond the potential complications, it is important to recognize that obesity can also limit surgical procedures, such as access to the cavity and the creation of stomas. Thus, a high BMI may be a limiting factor in surgical practice. For example, Maggioni et al. limited the indication for PE for patients with a BMI of less than 35 [5].

On the other hand, malnutrition and inadequate protein intake may also be predictive of adverse outcomes. Nielsen and colleagues, in a retrospective cohort of 195 patients undergoing PE, showed that patients with low albumin levels and a BMI of less than 25 were more likely to develop postoperative complications [25].

In addition to the physical criteria already mentioned, psycho-emotional and social aspects should also be taken into account when selecting a patient. PE is a morbid procedure associated with significant changes in the body and appearance of the individual, who must be oriented, prepared, and in agreement with the therapeutic proposals. The patient must also have a support network during the postoperative recovery period.

Table 1 provides an overview of the key parameters to consider when selecting a patient for pelvic exenteration.

### 2.2. Evaluation of Tumor Characteristics

After evaluating the criteria related to the patient’s physical and psychosocial health, it is important to analyze the tumor characteristics. Various aspects can be considered, such as histopathology, recurrence or disease-free interval, dimensions, and location of the tumor. Each of these will be described in more detail below and also summarized in Table 2.

Imaging studies may not be able to discern fibrosis secondary to radiotherapy from the persistence or progression of the disease. Therefore, histopathologic confirmation of the presence of the tumor is critical to the indication for PE.

Squamous cell carcinoma is the most common histologic type in cervical cancer patients undergoing PE. The second most common histology is adenocarcinoma. The oncologic outcomes when comparing these histologies are controversial in the literature. For most authors, Squamous cell carcinoma seems to present more satisfactory results, but without statistical relevance [10,11,26,27,28]. However, Baiocchi et al. found better results in patients with adenocarcinoma, in the context of PE for cervical and vulvar cancer [29].

Regarding histologic grade, grade 3 and undifferentiated tumors are associated with higher mortality [30]. Perineural invasion is associated with a higher risk of recurrence [9,30], and the presence of lymphovascular invasion is another finding associated with worse oncologic outcomes [9,31]. Rare histologies tend to have a worse prognosis and are usually not included in retrospective cohorts, so we do not have conclusive data on them.

The disease-free interval should be analyzed with caution because its impact on cancer outcomes is not well established. In some studies, patients with persistent disease or early recurrence have worse outcomes. McLean et al., for example, showed that patients with early recurrence, before completing 2 years of initial treatment, have lower overall survival (8 months) when compared to cases of late recurrence (33 months) [32]. In the Marnitz series, overall survival at 5 years was 16.8% for tumors that recurred before 2 years after primary treatment, 28% for cases between 2 and 5 years, and 83.2% after 5 years [14]. On the other hand, Straubhar et al., and also Westin et al., showed no statistically significant difference between the recurrence interval and oncological results [9,22]. We therefore believe that this is not a criterion that should be used in isolation when indicating PE.

The size of the tumor and its location are also essential for the indication of PE. Obtaining negative margins has been shown to be one of the main factors in increasing survival after PE [5,9,12,31,33,34]. Thus, the focus is on selecting cases where the R0 approach is feasible. Historically, tumors up to 3 cm located in the central region of the pelvis have been associated with better prognosis [5,21,35]. In Smith’s retrospective study of 151 women who underwent PE, tumors larger than 4 cm were associated with a higher risk of recurrence after the procedure. In addition, this analysis found a direct relationship between tumor size and positive margins; for every 1 cm increase in tumor size, the risk of positive margins increased by 11%. As expected, larger tumor size was also inversely associated with survival outcomes for gynecologic tumors [36].

In contrast, in the cohort study of Straubhar et al., most tumors selected for PE were greater than or equal to 3 cm in diameter, all had negative margins on histopathologic analysis (R0), and had no negative impact on survival [22]. This finding raises questions about the true impact of tumor size on survival and recurrence outcomes. Perhaps margin status is the real determinant of better outcomes after PE.

Recently, Martin et al. showed that the distance of the negative margin is also an issue to consider after PE. In a study of 124 patients who underwent the procedure, 80 had negative margins. These patients were then divided into two subgroups: (1) margins < 3 mm (close margins) and (2) margins ≥ 3 mm (distant margins). The overall survival results were 21 months for patients with positive margins and 62 months for negative margins. Considering negative margins, the overall survival was 32 months for close margins and 111 months for distant margins. Patients with distant negative margins had better oncologic outcomes than patients with positive margins. However, there was no difference in survival analysis between patients with positive and close negative margins [37].

In addition to tumor size, the extent of disease is another factor that affects the achievement of free margins. In the past, patients with tumors reaching the lateral region of the pelvis were not considered candidates for PE. Over the years and with the evolution of surgical techniques, complete resection of these lesions has become possible. The term “out-of-the-box surgery” was described by Caceres et al. in 2008 to refer to the extended resection of tumors that extend beyond the pelvic cavity [38]. Other terms such as extended pelvic resection (EPR), laterally extended endopelvic resection (LEER), and laterally extended pelvic resection (LEPR) are also used in the literature to refer to this approach [2,3,39].

A review on this topic was published by Daix and colleagues in 2022, clarifying the concept and potential indications for “out-of-the-box surgery” [40]. Several case series with such extended resections are available in the literature and the results vary as follows: R0 resection rate (75–99%); disease-free survival (34–75%); overall survival (34–75%); and postoperative complications (12–64%) [2,3,38,39].

Lymph node involvement, on the other hand, is associated with a worse prognosis as described by several authors [34,41,42,43,44,45]. In particular, in Schmidt’s series, there was no statistical difference in the overall survival of patients undergoing PE with pelvic lymph node involvement compared to cases without lymph node involvement. However, there was a significant decrease in the 5-year overall survival of patients with positive para-aortic lymph nodes (17%) compared to patients with positive pelvic lymph nodes only (45%) [11]. Thus, although not an absolute contraindication to PE, para-aortic lymph node involvement is associated with worse oncologic outcomes.

To conclude the issue of tumor location and extension, it is worth analyzing the evidence of small bowel involvement. Since 1964, Brunschwig warned of the difficulty of distinguishing tumor involvement of the small intestine from adhesions or inflammation. The author also described a series of cases in which resection of segments of the small intestine was performed in PE with satisfactory results [46]. Nowadays, in situations of carcinomatosis, with diffuse involvement of the serosa of the loops or even involvement of the mesentery, PE is contraindicated for curative purposes. However, if the tumor is directly involved, bowel resection may be considered [47]. Each case must be considered individually, as the anastomoses are associated with greater complications in the postoperative period.

**Table 2:** Evaluation of tumor characteristics—Key parameters.

Parameters	Recommendation
Histology type	The literature presents controversy regarding oncologic outcomes when comparing usual histologies
Histological features	Grade 3 and undifferentiated tumors are linked to higher mortality [30]. Perineural invasion is associated with an elevated risk of recurrence [9,30]. The presence of lymphovascular invasion is also connected with worse oncologic outcomes [9,31]
Disease-free interval	It’s impact on cancer outcomes remains inconclusive
Tumor size and location	Tumors up to 3 cm located in the central region of the pelvis have been associated with a better prognosis [5,21,35]
Tumor extension	Achieving negative margins has consistently been identified as one of the key factors enhancing survival after PE [5,9,12,31,33,34]. Even more extensive approaches may involve removal of lateral components of the pelvis, such as muscles, nerves, bones, and great vessels, characterizing extended pelvic resection or extended lateral endopelvic resection [2,3].
Lymph node involvement	It is associated with a worse prognosis [34,41,42,43,44,45].
Small bowel involvement	The presence of implants is an absolute contraindication to the procedure. However, resection due to direct invasion may be considered [47].

In order to define the location and extent of the tumor lesion, the information from the physical examination and the imaging studies should be used.

#### 2.2.1. Physical Examination

A general physical examination is important to determine the patient’s clinical status. For example, signs of anemia, lymph node enlargement, and cachexia should be evaluated.

A complete gynecologic examination, including a vaginal and rectal examination, can help assess the extent of the tumor. This analysis can identify parametrial extension, pelvic wall extension, and also vaginal wall involvement. In cases of advanced disease and complaints of pain, this may be performed under anesthesia. It is also through the physical examination that we can perform a biopsy to check for tumor persistence or recurrence.

#### 2.2.2. Imaging Tests

Imaging must be used to rule out the presence of distant metastases and to better define the location and extent of the tumor. These include computed tomography, magnetic resonance imaging (pelvis MRI), and PET-CT. MRI is an important image for assessing the tumor lesion and mapping the pelvis. It can also be used to assess the relationship between the tumor and local anatomical structures such as the pelvic wall, ureters, and nerves [48]. A study by Dresen et al. in patients with recurrent rectal cancer confirmed the importance of preoperative assessment with this imaging exam. In this analysis, an MRI was associated with a negative predictive value of 93–100% in the analysis of tumor invasion [49]. We believe that these data can be extrapolated in practice to gynecologic evaluation because they also represent persistent pelvic tumors.

PET-CT, on the other hand, is a tool that allows greater accuracy in detecting distant diseases, including extra-regional nodal involvement [50]. It can also be used to more accurately define tumor extent and differentiate pelvic fibrosis from viable tumors [51]. Invasive studies such as cystoscopy and rectosigmoidoscopy can be performed if there is a need to confirm invasion of adjacent organs such as the bladder and rectosigmoid.

### 2.3. Treatment Center Evaluation

In addition, for establishing operable and resectable cases, it is important to ensure that the treatment center has the minimum resources to perform the procedure. For example, the availability of a multidisciplinary team and imaging studies will influence the selection of cases. This is a radical and rare procedure that is usually not performed with high incidence even in the most specialized centers.

Matsuo et al. showed in their 2647 case series that the majority of PE procedures were performed in large treatment centers. These centers also had higher surgical complication rates, probably related to the more radical nature of the procedure, but lower mortality rates. Performing this procedure in large hospitals was also associated with lower mortality [13].

We believe that the success of PE is associated with the presence of an experienced team with the resources to select appropriate cases, provide preoperative preparation and care, ensure effective surgical technique, and reduce complication rates, as summarized in Table 3.

**Figure 2:**
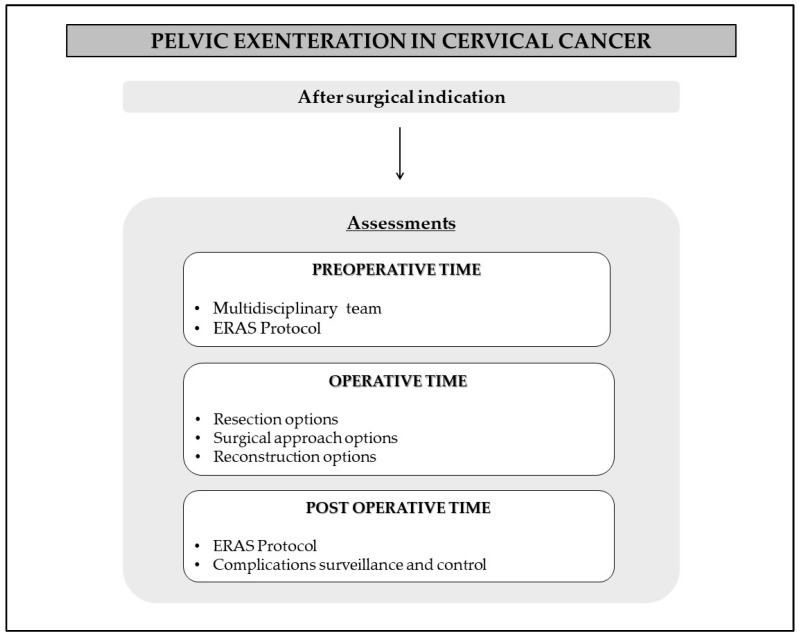
Recommendations for perioperative time.

After selecting patients who could benefit from PE with curative intent, the next step involves preparing for the procedure and surgical planning. The recommendations are summarized in Figure 2.

## 3. Preoperative Time

The importance of the multidisciplinary approach should be emphasized, with nutrition, psychology, nursing, physical therapy, and surgeons working together to achieve better surgical outcomes and fewer complications. Recommendations from the Enhanced Recovery After Surgery (ERAS) protocol can be used as a basis [52].

The ERAS protocol was developed in 2001 by a group of surgeons in Europe. Based on multidisciplinary care, its main objective is to improve postoperative outcomes, ensure early hospital discharge, and lower the rates of surgical complications. Some examples of measures recommended by the group are attention to the patient’s nutritional status, management of fluid administration, management of postoperative pain and nausea, early ambulation, prophylaxis of thromboembolic events, and prophylaxis of infection [52].

During this phase, it is crucial to correct hemoglobin levels in cases of anemia, enhance nutritional intake, psychologically prepare patients for a radical and aggressive procedure, and mark potential stoma sites. Specialized preoperative assessments, such as cardiology and pulmonology evaluations, may also be considered based on the patient’s comorbidities.

## 4. Operative Time

The surgical time can be divided into three steps: (1) resection of the tumor, (2) surgical approach, and (3) reconstruction procedures.

### 4.1. Resection of the Tumor

#### 4.1.1. PE Classification

PE can be named in different ways, depending on the location of the tumor and the organs involved in the en-bloc resection. In the traditional classification, the procedure that removes the uterus, uterine adnexa, parametrium, bladder, rectum, part of the vagina, and pelvic floor muscles is called total PE. Anterior PE spares the rectum, while posterior PE spares the bladder and urethra. In some cases, to obtain free margins, it may also be necessary to perform the perineal phase, with complete removal of the vagina, urethra, anus, and vulva, resulting in the formation of a pelvic cavity.

In 1990, Magrina proposed another form of subdivision to facilitate communication between healthcare teams and to standardize the technique. According to the author, PE can be divided into types I, II, III, and extended. In type I, tumor resection is performed above the supraelevator musculature, whereas in type II it is performed below this region. Type III is associated with vulvectomy, while extended involves an extension to the lateral region of the pelvis [53]. Regardless of the classification used, the central goal of curative PE is to remove the tumor lesion with negative oncologic margins (R0). As we have seen, this is one of the main determinants of oncologic outcome.

#### 4.1.2. Extended Resections

Extended resections may be indicated when the tumor has grown beyond the central region of the pelvis. Several studies have addressed this practice in recurrent or persistent pelvic and gynecologic tumors [54,55,56,57]. In these cases, en bloc removal of the tumor is indicated, potentially including the pelvic floor muscles, major vessels, nerves, and possibly bone tissue. A negative margin can be achieved in up to 75% of cases [54]. Data on the oncologic outcomes associated with this technique have already been presented in the case selection section, as we believe that this is a criterion that should really be considered before surgery.

In addition, it is essential to have a thorough knowledge of the pelvic anatomy and adequate vascular control when performing this procedure. Experienced surgeons from different fields should work together to achieve the best oncologic outcome and low complication rates.

For most authors, tumor involvement of the ischial foramen, external iliac vessels, and sacral nerve roots contraindicate PE with curative intent [12,13,47,55]. The sacrectomy procedure, for example, is described in the literature as an option for resecting tumors that affect this bone structure. In 2014, Milne et al. introduced a series of 100 cases of sacrectomies associated with PE in pelvic tumors [58]. Negative margins were achieved in 72% of cases, the overall survival rate was 38% and the disease-free survival rate was 30% at 5 years. Complication rates reached 74% and 6 patients had neurological damage. It should be noted, however, that this study involved mostly non-gynecological tumors, such as rectal or anal canal tumors [58].

Solomon and colleagues, in 2015, described a series of 200 cases of PE with extended resection [57]. Of the total, 132 patients underwent bone resections (sacrum = 107, ischium = 22, and pubis = 17). Additionally, of the total, only four patients had cervical cancer as an indication for the PE surgical procedure. We have no information on the correlation between cervical cancer and bone tissue excision in this study [57]. So, we understand that there is a paucity of data on bone resection for gynecologic tumors and even more so for cervical neoplasia. It appears to be a radical procedure that is not routinely indicated.

### 4.2. Surgical Approaches

The open approach is still considered the gold standard for performing PE. New surgical techniques have been developed with the intention of reducing morbidity and mortality and maintaining quality of life after radical surgery. This opens a discussion on the applicability of minimally invasive surgical techniques in performing this procedure.

The first complete laparoscopic PE for gynecologic malignancies was described by Pomel in 2003 [59]. Six years later, Lim described the first robotic approach [60]. Since then, other centers have begun to report their experiences, believing in the potential of the minimally invasive technique to reduce surgical complications and optimize postoperative recovery [61,62,63,64,65,66,67,68]. Until then, most of the available studies were a series of a few cases, with small sample sizes and no direct comparison with the laparotomic approach.

A systematic review published in 2018 aimed to compare outcomes between minimally invasive and open PE. Operative time, blood loss, margin status, morbidity and mortality rates 30 days after the procedure, and length of hospital stay were the outcomes analyzed. In this analysis, the minimally invasive technique was associated with lower blood loss, shorter hospital stays, and lower morbidity [69]. It should be noted, however, that this is an approach that should be carefully indicated, preferably in patients with favorable anatomy, and in front of an experienced team.

In a study published by Matsuo et al. in 2020, it was possible to retrospectively analyze a larger sample of patients and also compare the outcomes between the different surgical approaches. The study included 1376 women who underwent PE for gynecologic malignancies between October 2008 and September 2015, divided into two groups: minimally invasive (MIS) approach (*n* = 49) versus laparotomy (*n* = 1.327). The major outcomes included: lymphadenectomy rates which were similar between these groups; urinary tract diversion which was more frequent in the MIS approach; the incidence of a colostomy was higher in the open approach; no patient in the MIS approach received vaginal reconstruction; the incidence of bleeding was lower in the MIS approach; the MIS approach was associated with a lower incidence of serious complications such as shock, respiratory failure, sepsis, or thromboembolic events, but a higher chance of small bowel obstruction; and the MIS approach was associated with a shorter hospital stay and lower treatment costs [70].

In a recent publication, Sozzi et al. also reported shorter operative times, lower blood loss, and shorter length of hospital stay in laparoscopic surgery patients compared to laparotomy. According to the authors, laparoscopy allows better surgical results, with similar oncological outcomes [71].

Lampe and colleagues, in a review article about the limitations and opportunities of PE published in 2021, highlight what happened in the LACC trial and raise questions about the true oncologic safety of the minimally invasive approach in the context of persistent and advanced cervical disease [72]. Since 2018, with the publication of LACC, we have information that the minimally invasive approach has worse oncologic outcomes for the treatment of early-stage cervical cancer compared to the laparotomic approach [73].

Thus, the MIS approach is a promising technique with the potential to reduce the incidence of the numerous complications associated with PE. However, standardization of the surgical technique and clarification of indications are still needed for its consolidation. Accurate data on overall survival and disease-free survival must also be evaluated and studied in the long term.

### 4.3. Reconstruction Procedures

Once the tumor lesion has been removed en bloc, reconstruction of the intestinal tract, urinary tract, pelvic floor, and vagina may be considered. These procedures are generally aimed at restoring function and improving quality of life. However, the selection of cases and the technique used may also have a direct impact on the occurrence of complications. Urinary, intestinal, pelvic floor, and vaginal reconstructions are described below.

#### 4.3.1. Urinary Reconstruction

The first description of PE, proposed in the 1940s, involved en bloc resection of the pelvic organs as well as the vagina, vulva, and anus. The ureters were then implanted in the colon above the colostomy, forming a combined stoma. This shunt was associated with a high risk of urinary tract infection and hyperchloremic acidosis [1]. In recent decades, other reconstruction methods have been described with the aim of reducing complications and improving quality of life. The Bricker technique, described in the 1950s, reduced the rate of pyelonephritis, but patients were left with a wet ostomy after the ureters were implanted in the ileal conduit, potentially compromising their quality of life [74].

In an attempt to achieve urinary continence, several other approaches have been studied. A neobladder was then formed through various bowel segments, requiring periodic self-catheterization [75,76].

It should be added that the evidence in the literature on this subject is limited and there is no consensus on the superiority of any technique [77,78]. In fact, in a meta-analysis published in Cochrane that included five different studies, the authors suggest that multicenter studies with a larger sample size and random allocation should be performed [77].

#### 4.3.2. Intestinal Reconstruction

Terminal or lateral colonic anastomoses are feasible techniques to reconstruct intestinal transit after PE [79,80]. Ideally, a temporary ileostomy may be indicated as a form of protection in cases of dehiscence and inadequate healing of the anastomosis [79]. In cases of pelvic radiation, it is recommended to avoid low anastomoses [80]. If the anal sphincter region cannot be preserved, terminal colostomy is the procedure of choice [11,79,81].

#### 4.3.3. Pelvic Floor Reconstruction

The pelvic floor is a large cavity formed after total PE. It is an empty space that can be the focus of various complications such as the formation of collections, abscesses, and the prolapse of small bowel loops. Therefore, the filling of this region becomes a necessary practice to reduce the rate of complications. Among the different proposals described in the literature, the most common is the use of the omentum for local coverage. In the case of large defects, myofascial flaps such as the vertical rectus abdominis myocutaneous flap (VRAM) can also be used [10].

In a study published in 2006, Goldberg and colleagues described the various options for filling the pelvic void. The team believes in the superiority of VRAM as a reconstruction technique and has abandoned the use of synthetic forms or other materials to fill the pelvis [10].

#### 4.3.4. Vaginal Reconstruction

Vaginal reconstruction, or the development of a neovagina, is a complex procedure with potential complications. The goal is to restore sexual function and its indication must be individualized and decided with the patient. Matsuo’s case series presented a relatively low rate of vaginal reconstruction (22.3% of cases). The authors attribute this to the advanced age of most of the patients included in the study. In the same study, this procedure was considered an independent factor associated with the occurrence of surgical complications [13]. Various techniques are available, including skin grafts, myocutaneous grafts, and even tissue from intestinal loops, usually the sigmoid colon. In many cases, VRAM is chosen, which includes filling the pelvic cavity [10].

## 5. Perioperative Time

During surgery, different strategies can be used to minimize the occurrence of complications and to ensure a better oncologic outcome. Many of these are based on the ERAS protocol [52]. These recommendations include the use of combined epidural and general anesthesia for pain control, the use of elastic stockings and pneumatic compression boots to prevent thromboembolic events, adequate volume replacement, and the availability of blood products for hematometric and hemodynamic stabilization.

Postoperatively, we follow the same principles and the main goals are control of pain and vomiting, prophylaxis of thromboembolic events, nutritional support, hemodynamic and respiratory monitoring, and the early recognition of possible complications. Again, collaboration with professionals in the multidisciplinary team is essential.

In Matsuo’s retrospective cohort, evaluated between 2001 and 2018, the median length of hospital stay was 14 days. Over the years, there was a significant increase in the number of patients hospitalized for 28 days or more. The authors believe that this time may be related to an increase in the radical nature of procedures and, consequently, complications [13].

## 6. Complications

Throughout this article, we have often mentioned that PE is a morbid procedure. For this reason, it requires individualized indication and good preoperative planning.

The Clavien-Dindo classification can be used to stratify complications according to their severity and the intervention required. Complications can be graded from I to IV. Grade I, for example, represents changes that are not life-threatening and do not prolong hospital stay. Grade IV includes cases that result in death. Grade I and II complications are considered minor, while grade III and IV complications are considered major [82,83].

The general complication rates after PE described in the literature for gynecologic neoplasms range from approximately 25% to 94%, as presented in Table 4 [5,9,11,12,13,33,84,85,86,87,88]. If only major complications are considered, the rates range from 23% to almost 70% [11,85,86]. If we consider only cervical cancer, the complication rates range from 25 to 83.3% [11,84,88].

It should also be considered that the incidence of these adverse events may be higher in previously irradiated patients [35]. In their cohort of 2647 cases for gynecological cases, published in 2019, Matsuo et al. described 1802 patients (68.1%) who had postoperative complications. Of these, 1023 patients had multiple complications. The most common were bleeding, ileal or small bowel obstruction, surgical wound changes, respiratory failure, acute kidney injury, sepsis, thromboembolism, and pneumonia [13].

Over the years, there has been evidence of an increase in the number of complications following the practice of PE. Matsuo, for example, analyzed the evolution of outcomes of this procedure over 15 years, between 2001 and 2015 in the United States. The number of women with multiple postoperative complications increased significantly when comparing the period 2001–2005 to 2011–2015. For example, in the subgroup of patients with cervical cancer, this increase was approximately 68%. This is related to the higher prevalence of obesity in the population, as well as the increased indication for radical resection and extensive reconstruction [13].

On the other hand, with the improvement of technology and perioperative care, there has been a downward trend in postoperative mortality [13]. In studies published in the 1980s, for example, 6.3–7.2% of patients died postoperatively [21,89]. If we move to the 2000s, we find publications with surgical mortality rates close to 4% [90]. In 2019, Matsuo et al. showed a result of 1.9%, similar to another study published in 2011 with a rate of 2% [13,32].

## 7. Outcomes

### 7.1. Survival Analysis

The cancer outcomes studies available in the literature after PE are mostly retrospective cohorts with heterogeneous populations. Individual analyses for cervical cancer are rare, so we will also use data for pelvic neoplasms in general. Overall survival is defined as the time from exenteration to death. Table 5 lists the cohorts of patients who have undergone the procedure with their respective overall survival rates, which range from 22 to 70% [4,5,6,9,11,12,14,31,33,35,79,81,84,87,90,91,92,93,94]. Considering only cervical cancer, the 5-year overall survival rate ranged from 23.8 to 51% [6,11,14,84,92,93].

As mentioned at the beginning of this manuscript, there are many factors that affect cancer outcomes. Patient comorbidities, tumor characteristics, and compromised margins can have a direct impact on survival and recurrence rates.

### 7.2. Impact on Quality of Life

The analysis of quality of life (QoL) after PE depends on the surgical indication. For example, in patients with a palliative proposal, findings such as active bleeding, infection, tumor necrosis, and even fistulas are common. In this context, the goal of surgery is to improve the quality of life by controlling symptoms.

On the other hand, in curative indications, extensive resections may be associated with loss of function and changes in body image that negatively impact QoL. Expectations should be established with the patient as soon as the procedure is indicated, and psychological and emotional work should be encouraged. Knowledge of the possible outcomes of PE is important for the patient to make an informed decision.

Providing perioperative care as part of a transdisciplinary team reduces the negative impact on QoL. For example, recent studies have shown that lateral pelvic extension procedures may have a similar impact on patient QoL as traditional PE [95].

Vaginal reconstructions are also part of the process of restoring sexual function with a direct impact on quality of life. This is an issue that must be fully individualized with the patient.

## 8. Conclusions

PE is generally indicated as the last therapeutic option for patients with advanced or recurrent cervical cancer. The correct selection of cases is associated with a better oncological outcome and a lower rate of complications. The age of the patient and the time interval between primary treatment and recurrence should not be considered as exclusion criteria in isolation. Tumor extension to the lateral pelvic region and pelvic lymph node involvement are no longer absolute contraindications to the procedure. Over the years, mortality rates have decreased, and overall survival has reached values close to 50% at 5 years, making it a possible curative procedure. However, complications are still common and potentially serious. Because of the impact on quality of life, the procedure should always be individualized and considered by the patient and the multidisciplinary team.

## Figures and Tables

**Table 1:** Assessment of the patient’s clinical condition—Key parameters.

Parameters	Recommendation
Age	Chronological age is not a limiting factor for PE [7,14,19,20,21]
Comorbidities	Directly associated with postoperative adverse events, longer hospital stays, and higher mortality [13]
BMI	Obesity is directly linked to a higher incidence of postoperative complications [24], just like malnutrition [25]
Psycho-emotional and social aspects	The patient must be informed, prepared, and in agreement with the therapeutic proposals

**Table 3:** Treatment center evaluation—Key parameters.

Parameters	Recommendation
Treatment center	The team’s experience and the resources provided are associated with the success of the treatment

**Table 4:** Perioperative morbidity and mortality after pelvic exenteration.

Authors	Number of Patients	Cancer	Morbidity	Mortality
Maggioni et al., 2009 [5]	106	Gynecologic	66%	0% (30 days)
Fotopoulou et al., 2010 [85]	47	Gynecologic	70.2 (major)	8.5%
Benn et al., 2011 [12]	54	Gynecologic	50% (early)	-
			61% (late)	
Schmidt et al., 2012 [11]	282	Cervical	51%	5%
Westin et al., 2014 [9]	160	Gynecologic	94.4%	1.3%
Matuso et al., 2019 [13]	2647	Gynecologic	68.1%	1.9%
Tortorella et al., 2019 [86]	138	Gynecologic	67%	2.2% (90 days)
Lewandowska et al., 2020 [88]	44	Cervical	25% (major)	2.3%
Ter Glane et al., 2021 [33]	47	Gynecologic	80.9%	4.3%
Ter Glane et al., 2022 [84]	24	Cervical	83.3%	4.2%
Haidopoulos et al., 2022 [87]	138	Gynecologic	12.5% (major)	2.2%
			42% (grade 2+)	

**Table 5:** Survival following pelvic exenteration.

Authors	Number of Patients	Cancer	Overall Survival
Berek et al., 2005 [90]	75	Gynecologic	54% in 5 years
Marnitz et al., 2006 [14]	55	Cervical	36.8% in 5 years
Maggioni et al., 2009 [5]	106	Gynecologic	31.6% in 5 years
Benn et al., 2011 [12]	54	Gynecologic	34% in 50 months
			22% in 100 months
Kaur et al., 2012 [79]	36	Gynecologic	44% in 5 years
Schmidt et al., 2012 [11]	282	Cervical	41% in 5 years
Haidopoulus et al., 2012 [87]	138	Gynecologic	44.6% alive in 38.84 months
Jager et al., 2013 [81]	28	Gynecologic	70% in 5 years
Chiantera et al., 2014 [6]	167	Cervical	38% in 5 years
Westin et al., 2014 [9]	160	Pelvic	40% in 5 years
Lago et al., 2019 [4]	23	Gynecologic	41.6% in 5 years
De Gregório et al., 2019 [31]	37	Gynecologic	46.4% in 5 years
Ter Glane et al., 2021 [33]	47	Gynecologic	21.3% in 3 years *
Gheorghe et al., 2021 [91]	23	Pelvic	66% in 5 years
Stanca et al., 2022 [92]	47	Cervical	48.7% in 5 years
Ter Glane et al., 2022 [84]	24	Cervical	23.8% in 5 years
Bouraouri et al., 2022 [93]	41	Cervical	51% in 5 years
Sibiani et al., 2022 [94]	277	Gynecologic	32.3% in 5 years
			36.8% in 3 years
Moolenaar, et al., 2023 [35]	90	Gynecologic	51.1% in 2 years

* Includes metastatic cases.

## Data Availability

The data presented in this study are available in this article.
